# GC-MS analysis and nutra-pharmaceutical potential of *Mentha piperita* essential oil extracted by supercritical fluid extraction and hydro-distillation

**DOI:** 10.1016/j.heliyon.2024.e35282

**Published:** 2024-07-26

**Authors:** Ali Abbas, Farooq Anwar, Naveed Ahmad, Afifa tur Rehman, Osama A. Mohammed, Mustafa Ahmed Abdel-Reheim, Munawar Iqbal, Shahid Iqbal, Arif Nazir

**Affiliations:** a Institute of Chemistry, University of Sargodha, Sargodha, 41000, Pakistan; b Department of Chemistry, Govt. Islamia Graduate College, Chiniot, Pakistan; c Department of Food Science, Faculty of Food Science and Technology, Universiti Putra Malaysia, 43400, UPM Serdang, Selangor, Malaysia; d Department of Chemistry, Division of Science and Technology, University of Education, Lahore, Pakistan; e Institute of Molecular Biology and Biotechnology, University of Lahore, Sargodha Campus, Sargodha, Pakistan; f Department of Pharmacology, College of Medicine, University of Bisha, Bisha, 61922, Saudi Arabia; g Department of Pharmaceutical Sciences, College of Pharmacy, Shaqra University, Shaqra 11961, Saudi Arabia; h School of Chemistry, University of the Punjab, Lahore, Pakistan; i Department of Chemistry, The University of Lahore, Lahore, Pakistan; j Department of Pharmacology and Toxicology, Faculty of Pharmacy, Beni-Suef University, Beni Suef 62521, Egypt

**Keywords:** *Mentha piperita* L., Essential oil, Menthol, Bioactivity, GC-MS

## Abstract

This study reports the comparative evaluation of yield, physico-chemical composition and biological attributes (antioxidant activity, antimicrobial activity, biofilm inhibition and hemolytic activity) of peppermint (*Mentha piperita* L.) essential oil (EO) obtained by hydro-distillation (HD) and supercritical fluid (CO_2_) extraction (SCFE) methods. The yield (%) of EO obtained by HD (0.20 %) was significantly (*p* < 0.05) higher than that of SCFE (0.13 %) while the variation in the physical parameters like solubility, color, density (at 25 °C) and refractive index (at 25 °C) was not significant between the tested oils. The data of chemical compositional analysis revealed that menthol was the key component in the EO obtained by HD (52.85 %) and SCFE (45.51 %), followed by menthone [HD (25.93 %) and SCFE (27.3 %)] and eucalyptol [HD (8.59 %); SCFE (8.92 %)]. The EO extracted with supercritical fluid (SCFE-EO) exhibited superior (*p* < 0.05) DPPH free radical inhibition potential (52 %) with an IC_50_ value of 15.65 μg/mL and reducing power compared to that of HD-EO. The highest antimicrobial activity was exhibited by SCFE-EO against *Pasturella multocida* with an inhibition zone of 18.00 mm (MIC value of 86 μg/mL). The results of biofilm inhibition and hemolytic activity revealed that the SCFE method was superior to recover high quality EO in comparison to the HD method. The peppermint EO obtained by SCFE, owing to potent bioactive components, can be a potential candidate to develop nutra-pharmaceuticals.

## 1 Introduction

Plants, as a promising source of physiologically active compounds, have been used as folk medicines in different civilizations of the world. A variety of modern drugs have been obtained from various parts of plants (flowers, roots, leaves, roots, stem, bark and fruits) to treat infectious diseases [1,2]. Currently, there is an increasing trend for utilizing plants and herbs as a natural source of medicines due to their safer nature, and the health implications of pharmaceutical drugs [3,4]. In fact, in line with the current developments of optimal nutrition, the plant-derived natural products and phytomedicines are gaining high recognition for maintaining good human health and well-being [5,6].

*Mentha* is a medicinally popular and economically important genus with around 30 species that have a characteristic aroma, and can grow in diverse agro-ecological conditions the world over [7]. Almost all the species of *Mentha* are used as food flavoring agents in different parts of America, Australia, Europe and the Middle East [8]. They are also used as natural ingredients in different foods items such as salads, bread, herbal teas and soups [9] and herbal cosmetics [10]. The extracts and essential oil (EO) obtained from *Mentha* species are also employed as traditional medicine for the treatment of different health disorders [7] by exhibiting multiple therapeutic benefits which can be linked to the availability of potent bioactives [11].

*Mentha piperita*, also called as “peppermint”, is the hybrid species obtained by crossing *Mentha aquatica* and *Mentha spicata* L [12]. Peppermint is widely cultivated for its medicinal, pharmaceutical and food flavoring applications. In the United State peppermint is mainly grown to produce EOs [13]. It has a broad-spectrum medicinal application and is used in the formulation of traditional medicines for its uses in toothpaste, mouthwashes, aromatherapy, and in topical preparations to cure irritation and inflammation [14]. Its fresh leaves are used to garnish the food, production of margarita drinks, refreshing tea and as a flavoring agent for chocolates, desserts, and ice cream [15].

Peppermint EO is used as a traditional medicine to treat muscular pain, menstrual cramps and rheumatism [8]. It has also been reported to give comfort in health conditions such as heartburn, digestion disorder, flatulence, and nausea [16]. It possesses a strong menthol smell and shows biological activities like antioxidant, antimicrobial and insect-repellant activities [14]. Menthol, the major component of peppermint EO, is used as an antiseptic agent as well as in the formulations of local anesthetics. Due to its refreshing menthol odor and appreciable antimicrobial and antioxidant activities, support its wide-scale usage as preservative food and pharmaceutical preparations [8]. The qualitative and quantitative profile of EOs is reported to be associated with the nature of plant materials and extraction techniques employed [5,17]. Improper extraction methods can damage or change the composition of EO resulting in the loss of its food flavoring and intrinsic biological characteristics [18]. Furthermore, the use of different solvents, and extraction conditions (temperature, pressure and time) can lead to produce EOs with varied bioactives concentration biological potential [5]. Conventionally, hydro-distillation, and steam distillation [19] are mostly employed to extract EO from plant material but nowadays, a modern technique, supercritical fluid extraction (SCFE) is also gaining popularity to obtain the volatile constituents from different plant materials [20]. In the SCFE technique, normally liquid CO_2_ is used as supercritical fluid due to its no toxicity and ease of removal due to its volatility [5].

Pakistan is an agricultural state and is blessed with a rich biodiversity and natural flora, where various plants are being grown in different parts of the country including hilly areas, deserts and plains. The Soon Valley, District Khushab, Punjab, Pakistan has specific agro-climatic conditions with low annual precipitation while its temperature ranges from 1 °C to 36 °C (January to June). Some medicinal plants from this particular region have been documented to possess a unique phytonutrients profile [20,21]. Peppermint has been extensively used by the local population so there is a need for a systematic study to compare the effect of different extraction techniques on the yield and biochemical composition of EO from this species belonging to less explored areas aiming to highlight its potential utilization as an ingredient of functional food and pharmaceuticals. Therefore, the current research was focused on the appraisal of comparative compositional analysis and biological attributes of peppermint (*Mentha piperita*) EOs, extracted by HD and SCFE, from this specific area (Soon Valley) of Pakistan leading to explore nutra-pharmaceutical prospects of this species.

## 2 Material and methods

### 2.1 Collection and pretreatment of samples

Aerial parts of the plant (leaves and stem) of peppermint (*Mentha piperita*) were harvested during the spring season (April 2020, Voucher ID: UE-2245**)** from Soon Valley, District Khushab, Punjab, Pakistan. The samples were authenticated by a botanist from the Department of Botany, University of Education, Lahore. These plant samples were washed to remove the associated debris (if any) and dried in the shade till the moisture content was achieved at 6.5 %.

## 3 Extraction of peppermint EO

### 3.1 Two different techniques (HD and SCFE) were utilized to recover EO from the dried sample matrices

#### 3.1.1 Hydro-distillation

A Clevenger-type apparatus (MediLab, India, sample capacity: 3 kg) was used to extract the EO. In this method the sample was hydro-distillated for 3 h and the extracted EO was dried using anhydrous Na_2_SO_4_ to store at –4 °C till further analysis [8,22].

#### 3.1.2 Supercritical fluid extraction

For the extraction of peppermint EO by SCFE, a SCF extractor, commercial scale (DEVEN, supercritical Pvt. Ltd., India), was used. The extractor was pre-heated at 45 °C for 60 min and then 5 kg of sample was fed into it, the temperature of the extractor was maintained and liquid CO_2_ was allowed to interact with the plant sample at the flow rate of 10 mL/min maintaining the inflow pressure of 100 bar. The static extraction was performed for 90 min and then processed for dynamic extraction for 30 min. Consequently, obtained extracts were collected in a glass vial collector and preserved for further analysis [20,28]. The same experimental conditions, including temperature and pressure were maintained for replicate analysis to reproduce the results with high accuracy.

### 3.2 Physical analysis

The solubility test of the EO recovered with both techniques was performed in 70 % alcohol. The density (25 °C) and refractive index (25 °C) were determined [23] using digital densimeter (DMA-602, Austria) and refractometer (RX-7000, Atago, Japan), respectively. Each analysis was performed in triplicates and the data thus produced was averaged.

## 4 Chemical composition of EO

### 4.1 Gas chromatography (GC)-FID

The composition analysis of peppermint EO was performed using a gas chromatograph (PerkinElmer, 8700, USA). Separation of volatile components was carried out using an instrument attached to a capillary column with the following dimensions (HP-5MS, 30 m × 0.25 mm) followed by detection by a flame ionization detector (FID). The column temperature was programmed in such as way that the initial temperature (80 °C) was maintained for 3 min and then increased to 220 °C at a ramp rate of 4 ^°^C/min and finally maintained for 10 min. The injector was adjusted at 220 °C to inject the sample in split mode, while the temperature of 290 °C was maintained for the detector. The carrier gas (helium) flow rate was optimized to be 1.5 mL/min. The quantification was performed with the built-in software of the system. The same conditions like, the flow rate of carrier gas, temperature programming and detector conditions, were maintained for replicate analysis to achieve high reproducibility.

### 4.2 GC-MS analysis

The composition of the extracted EOs by both techniques was further authenticated by Gas chromatography (Agilent, 6890N, USA) equipped with a mass spectrometer (MS-5957). The EO was injected using an autosampler (7683-B). The conditions of the gas chromatography used were similar to those described earlier in the GC-FID method. The MS transfer line temperature was 290 °C, while the energy of electron ionization mode was set to 70 eV for MS detection with a scanning range varying over 50–600 *m*/*z*. The same experimental conditions were maintained for reproducibility of the data during replicate measurements.

#### 4.2.1 Components identification

The separated compounds were identified by following the procedure previously adopted by Abbas et al., 2017 [5] by finding out their RI (retention indices) relative to *n*-alkane (C_9_ – C_24_) and comparing it with the literature. The obtained results were further authenticated by comparing their data obtained by a mass spectrometer with the library of NIST mass spectra and literature.

### 4.3 Antioxidant activity assays

#### 4.3.1 DPPH^.^ Radical scavenging activity

The DPPH radical scavenging ability of investigated oils was estimated following a method reported by Bozin et al., 2006 [24]. Different concentrations (0.5–100 μg/L) of EO were added into 1 mL of 90 μM DPPH radical solution, then added into methanol (95 %) to make the final solution (4.0 mL) followed by incubation for 60 min and then absorbance of the solution was observed at 515 nm. Butylated hydroxytoluene (BHT) was processed as positive control separately and the inhibition potential (I %) was calculated as:

$$I\;\left(\%\right)=100\;x\;\left[{\left(A_{b}–A\right)}_{s}/A_{b}\right]$$

A_s_ = absorbance of the tested sample.

A_b_ = absorbance of control mixture.

The IC_50_ value (50 % inhibition concentration) was calculated by a graph between sample concentration and percent inhibition.

#### 4.3.2 Reducing power

The assay to estimate the antioxidant activity by finding out the reducing power was determined using a protocol described by Abbas et al., 2017 [5]. Briefly, a buffer (Na-phosphate, 0.2 M, pH 6.6, 5.0 mL) and K_3_[Fe(CN)_6_] solution (5.0 mL, 1.0 %) were mixed with different concentrations (2.5–10.0 mg) of the tested sample, separately. Five milliliters of C_2_HCl_3_O_2_ solution (10.0 %) was reacted with this solution after incubation at 50 °C. The resulting solution was spun at 980×*g* and supernatant (5.0 mL) was taken to mix with distilled water (5.0 mL) then finally allowed to react with 1.0 mL of FeCl_3_ solution (0.1 %). The absorbance of the final solution was recorded at 700 nm.

### 4.4 Antimicrobial activity

Antimicrobial properties of tested EOs were determined against infectious bacterial (*Staphylococcus aureus*, *Escherichia coli*, *Pasturella multocida* and *Bacillus subtilis*) and fungal (*Alternaria alternata*, *Ganoderma lucidum*, Aspergillus niger and *Aspergillus flavus*) strains. These microbial strains were cultured on nutrient agar (for bacteria) (Oxoid) and dextrose agar (for fungus) (Oxoid) for optimum growth at the temperature of 37 °C and 28 °C, respectively.

#### 4.4.1 Measurement of inhibition zone (mm) using disc diffusion method

This method was used to estimate the zone of inhibition [25]. Microorganisms were inoculated on an agar plate and the sterilized filter paper disc (6.0 mm), soaked in the tested sample, was placed on it. Streptomycin (30μg/disc) and fluconazole (30μg/disc) were processed as a positive control for antibacterial and antifungal activity, respectively, while the negative control was also run using a blank disc without any sample. After incubation, for bacteria at 37 °C and fungus at 28 °C, the zone of inhibition (mm) was measured to calculate antimicrobial activity.

#### 4.4.2 Minimum inhibitory concentration (MIC)

MIC value was calculated using microdilution broth assay [25] was performed to determine the MIC value of tested EO. In this method, a series of sample dilutions were mixed with 160 μL of each nutrient broth (NB) and sabouraud dextrose broth (SDB) for bacterial and fungal strains in a 96-well microtiter plate. Sterility control and growth control were also processed along with sample treatment. The microorganism broth culture (20 μL; 5 × 10^5^ CFU/mL) was inoculated followed by incubation at 37 °C (for bacterial strains) and at 28 °C (for fungal strains).

### 4.5 Biofilm inhibition

The tested EO was evaluated for biofilm inhibition potential according to a procedure used by Abbas et al., 2017 [5]. The cell suspension (100 μL) of different bacterial strains, including *E.coli* and *S.aureus*, was mixed with tested EO in a microtiter plate. After incubation (37 °C) liquid suspension was removed and the residual pellet was stained with crystal violet solution (1.0 %) followed by washing with distilled water and then with ethanol solution (95.0 %). After incubation, absorbance was recorded at 570 nm to calculate inhibition of biofilm (%) formation as:

$$Biofilm\;inhibition\;\left(\%\right)=\frac{A_{control}-A_{treatment}}{A_{control}}\times 100$$

### 4.6 Hemolytic activity

Hemolytic activity of oil samples was determined by using a method used by Malagoli, 2007 [26]. During this *in-vitro* analysis, the samples of blood from volunteers were centrifuged for 5 min at 5000 rpm and reconstitute blood in phosphate buffer to prepare its solution (2.0 %) of erythrocyte. Various concentrations (50–500 μg/mL) of the tested sample were separately mixed with saline solution and erythrocytes suspension (2.0 %) followed by incubation at 25 °C. Centrifuged the reaction mixture to isolate supernatant for the estimation of secreted hemoglobin by taking absorbance at 540 nm. During the experiment, the positive control (0.1 % Triton X-100) and negative control (without sample) were also used along with samples under the same set of experimental conditions. The hemolytic activity was calculated as:

$$Percentage\;hemolysis\;\left(\%\right)=\frac{Absorbance\;of\;sample}{Absorbance\;of\;positive\;control}\times 100$$

### 4.7 Statistical analysis

The results of all analyses (conducted in triplicates) were analyzed by ANOVA (STATISTICA, Stat Soft Inc., USA) [27].

## 5 Results and discussion

### 5.1 Yield percentage and physicochemical parameters

The result of percentage yields and physicochemical parameters are tabulated (Table 1). The yield (0.13 %) of SCFE-EO was significantly (*p* < 0.05) lower than HD-EO (0.2 %). This variation in the extraction yield of EO can be ascribed to different factors including variety, nature and maturity stage of the plant material and the conditions of extraction [28]. The lower yield of SCFE-EO might be due to the selectivity and specificity of the technique employed, along with the polarity and chemical nature of the solvent used for the extraction of EO. The temperature of the extraction method is another key factor that contributes to changes in the yield and composition of EO from plant material. The higher yield of EO using HD might be due to the effective recovery of terpenoids. The result of the percentage yield in the current study is in line with the literature [5,28,29].

**Table 1:** Yield (%) and physical analysis of *Mentha piperita* EOs extracted by different methods.

Technique	Density g/mL (25 °C)	Refractive index (25 °C)	Color	Yield (%)	Solubility
SCFE	0.931 ± 0.02^a^	1.459 ± 0.04^a^	Yellowish Brown	0.13 ± 0.01 ^b^	Soluble in 2.3 volume of 70 % alcohol
Hydro-distillation	0.916 ± 0.03^a^	1.428 ± 0.04^a^	Light Yellow	0.20 ± 0.02^a^	Soluble in 2.9 vol in 70 % alcohol

The result is presented as the mean of three replicate measurements ± standard deviation. The significant difference (*p* < 0.05) between the two extraction techniques is represented by superscripts within the same column.

The intensity of color of EO showed a large difference from yellowish brown (SCFE-EO) to light yellow (HD-EO). The solubility (2.3 volume of 70 % alcohol) of SCFE-EO was a lower than the solubility (2.9 volume of 70 % alcohol) of HD-EO. The refractive index (25 °C) of EO extracted by the SCFE (1.459) was higher than that of HD (1.428), however, this is not a significant difference. Likewise, the density (25 °C) of SCFE-EO was a little higher (0.931 g/mL) than that of HD (0.916 g/mL). The minor change in the physical parameter of the tested EO might be linked to composition and methods of extraction [28,30].

### 5.2 Chemical composition

**Fig. 1:**
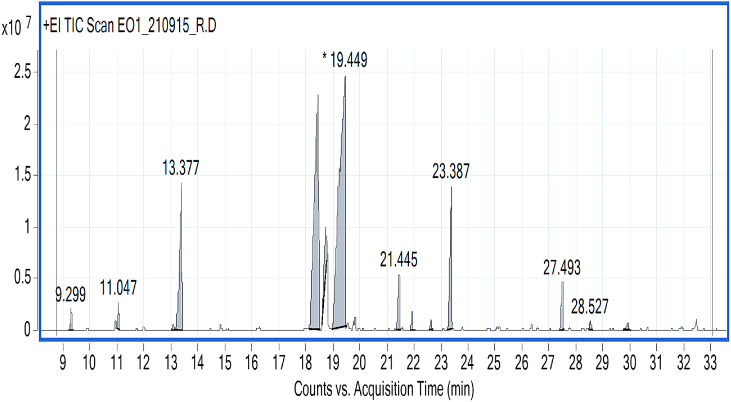
GC-MS chromatogram of EO obtained by SCFE from *Mentha piperita*.

**Fig. 2:**
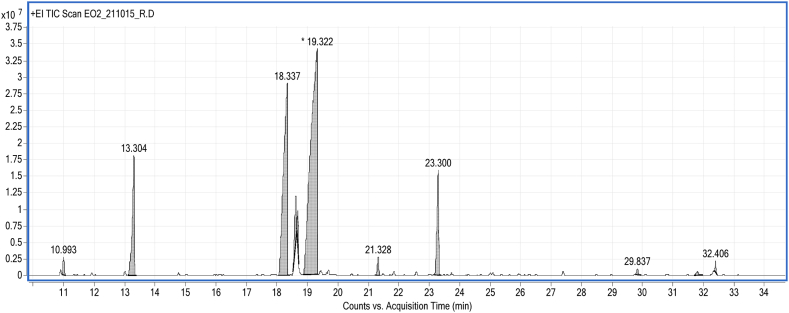
GC-MS chromatogram of EO obtained by HD from *Mentha piperita*.

The chemical composition of the tested EO obtained by SCFE and HD was analyzed using GC-FID/GC-MS. The GC-MS chromatogram relating to the analysis is presented in Fig. 1, Fig. 2, while the percentage composition of the major components is given as Table 2. The results show that total 14 components were identified in the EO obtained by SCFE with menthol (45.51 %) as the major component followed by menthone (27.3 %), while in the HD-EO, total 12 components were identified where menthol (51.85 %) was also dominated followed by menthone (25.93 %).

**Table 2:** The chemical composition (%) of some major components in *Mentha piperita* EOs extracted by different methods.

Sr. #	Name of Compound	Retention index	SCFE	Hydro-distillation	Identification Method
1	Eucalyptol	1033	8.92 ± 0.43^a^	8.59 ± 0.43^a^	x, y, z
2	Menthone	1154	27.3 ± 1.12^a^	25.93 ± 1.14^b^	x, y, z
3	trans-Menthone	1161	3.11 ± 0.18^a^	2.56 ± 0.08^a^	x, y, z
4	Menthol	1170	45.51 ± 1.93^b^	51.85 ± 2.16^a^	x, y, z
5	Menthyl acetate	1297	7.28 ± 0.34^a^	6.67 ± 0.32^a^	x, y, z
6	Caryophyllene	1425	2.00 ± 0.11	–	x, y, z
**Total components identified**			14	12	–
**Total Percentage of Identified Compounds**			98.64 %	99.32 %	–

Result is presented as mean of three replicate measurements ± standard deviation. The significant difference (*p* < 0.05) between two extraction techniques is represented by superscripts within the same row.

x = Retention index.

y = Comparison with authentic compounds.

z = Comparing mass spectra.

Literature reports reveal the presence of oxygenated monoterpenes in the EO obtained from *Mentha piperita*, including menthol, eucalyptol, menthone, menthyl acetate and menthofurone [31,32]. Two major components in this study, menthol and menthane were found to be present in peppermint EO over the range of 10 %–63 % and 12 %–76 %, respectively which are in line with the literature [33]. In another report [34], 23 compounds were identified in peppermint EO where menthol (38.33 %) and menthone (21.45 %) were noted to be the principal components. Furthermore, İşcan et al. (2002) [35] also reported the presence of menthol (28–42 %) and menthone (18–28 %) as same principal compounds in peppermint EO.

Similarly, in another recent published study, menthol (47.0 %) and menthone (23.1 %), along with other menthol derivatives (neomenthol 3.6 % and menthofurane 3.7 %) were found to be the major components in *M. piperita* EO [36]. According to a latest study by Olayemi et al. (2024) [37], the major compound in peppermint EO was menthol (40.4 %) followed by menthone (12.3 %).

### 5.3 Antioxidant activity

The reducing power and free radical scavenging capacity were estimated to assess antioxidant potential of the tested oils.

DPPH is a free radical which accepts a proton from reducing agents (bioactives) to change its violet color to yellow. The DPPH free radical potential was enhanced as a function of increasing concentration of antioxidant compounds [5,38]. The results of this assay is presented as Table 3. It can be noted from the result that the radical scavenging activity of SCFE-EO was quite higher (52 %) with IC_50_ (15.65 μg/mL) than HD-EO (43 %) with IC_50_ (13.48 μg/mL). The EOs recovered from many odiferous plants possess antioxidant activity to scavenge free radicals and thus contribute to retarding the process of oxidation in living organisms, therefore these plants can be used to treat degenerative and cardiovascular diseases [20,39].

**Table 3:** Antioxidant activity of *Mentha piperita* EOs extracted by different methods.

Extraction Technique	DPPH Radical Scavenging Assay		Reducing Power			
			Concentration (mg/mL)			
	Inhibition (%)	IC_50_ (μg/mL)	2.5	5.0	7.5	10.0
**SCFE**	52 ± 3^a^	15.65 ± 0.48^a^	0.78 ± 0.08^a^	0.94 ± 0.08^a^	0.98 ± 0.10^a^	1.24 ± 0.12^a^
**Hydro- distillation**	43 ± 2 ^b^	13.48 ± 0.32 ^b^	0.69 ± 0.03^a^	0.92 ± 0.06^a^	1.03 ± 0.10^a^	1.13 ± 0.12 ^b^
**BHT**	–	10.21 ± 0.29^c^	–	–	–	–

Result is presented as mean of three replicate measurements ± standard deviation. The significant difference (*p* < 0.05) between two extraction techniques is represented by superscripts within the same column.

Reducing power of plant EO is another parameter to check its antioxidant potential [40]. The color intensity of the solution is directly correlated with reducing ability of the tested EO. There is a regular increase in the absorbance, hence reducing power, with an increase in the EO concentration. SCFE-EO was found to exhibit higher reducing power (1.24) as compared to HD-EO (1.13).

In a related study, *M. longifolia* EO was found to express good antioxidant and free radical scavenging potential (IC_50_, 0.66 ml/ml of solution) [41]. Another relative study, *M. piperita* EO exhibited better free radical scavenging activity compared to *M. aquatica* and *M. longifolia* [42]. *M. longifolia* EOs displayed considerable antioxidant capacity due to the occurrence of carvone and polyphenols [43]. Likewise, Mentha EOs were also found to represent substantial potential to inhibit the formation of peroxides in lipids and good DPPH scavenging ability due to the presence of phenolics and flavonoids [44].

### 5.4 Antimicrobial activity

Antimicrobial activity was determined by evaluating tested EOs against a set of pathogenic bacterial cultures including *S.aureus, E.coli, P.multocida* and *B.subtilis* and fungal; stains such as *A. alternata, G. lucidum*, A. niger and *A. flavus* (Table 4). The highest antibacterial activity was shown by SCFE-EO (inhibition zone 18.00 mm) against *P.multocida* indicating minimum inhibitory concentration (MIC) of 86 μg/mL. Meanwhile, the HD-EO exhibited maximum antibacterial activity (inhibition zone 14.00 mm) against *E. coli* with MIC value of 102 μg/mL. The minimum antibacterial activity was exhibited by SCFE-EO (Inhibition zone 11.00 mm) against *S.aureus* with MIC value 109 μg/mL and HD-EO showed minimum antibacterial activity (inhibition zone 11.00 mm) against *B. subtilis* with MIC value 114 μg/mL. Overall, the peppermint EO exhibited lower antibacterial activity when compared to the standard drug (Streptomycin).

**Table 4:** Antimicrobial activity of *Mentha piperita* EOs extracted by different methods.

Microorganisms	Inhibition Zone (mm)			Minimum Inhibitory Concentration (μg/mL)		
	SCFE	HD	*Standard Drug*^a^	SCFE	HD	*Standard Drug*^a^
**Bacteria**						
*S.aureus*	11.00 ± 0.34 ^b^	12.00 ± 0.28^a^	28.00 ± 1.12	109 ± 3 ^b^	116 ± 5^a^	62 ± 2
*B.subtilis*	12.00 ± 0.31^a^	11.00 ± 0.27 ^b^	31.00 ± 1.18	121 ± 5^a^	114 ± 4 ^b^	73 ± 3
*P.multocida*	18.00 ± 0.41^a^	13.00 ± 0.29 ^b^	30.00 ± 1.12	86 ± 3 ^b^	118 ± 4^a^	54 ± 2
*E.coli*	15.00 ± 0.32^a^	14.00 ± 0.41 ^b^	29.00 ± 1.21	98 ± 3 ^b^	102 ± 3^a^	63 ± 3
**Fungus**						
*G.lucidum*	13.00 ± 0.56 ^b^	15.00 ± 0.49^a^	26.00 ± 1.08	113 ± 3^a^	97 ± 3 ^b^	73 ± 2
*A.flavus*	12.00 ± 0.32 ^b^	14.00 ± 0.42^a^	28.00 ± 1.15	121 ± 5^a^	114 ± 4 ^b^	78 ± 2
*A.niger*	15.00 ± 0.53^a^	14.00 ± 0.47^a^	30.00 ± 1.23	104 ± 3 ^b^	112 ± 4^a^	53 ± 2
*A.alternata*	11.00 ± 0.29 ^b^	13.00 ± 0.38^a^	24.00 ± 1.02	123 ± 4^a^	106 ± 4 ^b^	83 ± 3

Result is presented as mean of three replicate measurements ± standard deviation. The significant difference (*p* < 0.05) between two extraction techniques is represented by superscripts within the same row.

a *Streptomycin* for bacterial strains and *Fluconazole* for fungal strain.

SCFE-EO showed the highest antifungal activity (inhibition zone 15.00 mm) against *A. niger* with MIC value at 104 μg/mL while HD-EO was found to express superior antifungal potential (inhibition zone 15.00 mm) against *G. lucidum* with MIC of 97 μg/mL. The EOs obtained by both the techniques showed lower antifungal capacity compared to that of standard drug (Fluconazole).

Different infectious diseases are predominantly caused by several pathogenic bacterial and fungal strains [45]. Due to the perceived toxicity of synthetic drugs, phyto-medicines have been found to be safer and effective to treat infectious diseases [4,28]. Anwar et al. [43] found that *Mentha longifolia* essential oil (EO) had strong antibacterial activity against various bacterial strains. However, the antimicrobial ability of the EO was influenced by its chemical contents. In another study, SCF- extracted EO from *Mentha spicata* leaves showed strong antibacterial activity against different bacterial strains (Saba & Anwar, 2018). According to Soković et al., 's 2009 study [46], there was a notable antifungal effect of the essential oils derived from *M. piperita* and *M. spicata* against seventeen distinct pathogenic fungal strains.

### 5.5 Biofilm inhibition and hemolytic activity

The biofilm inhibition potential of tested peppermint EO was evaluated against the different strains of *S.aureus* and *E.coli* (Table 5). The maximum biofilm inhibition was exhibited by SCFE-EO (74.57 %) against *E.coli*, however this potential was found to be lower than that of standard drug (Rifamacin) in this experiment. Few antioxidants have been shown to damage hemoglobin and be unsafe for continuous usage. Thus, before EO is suggested for phytomedicine and nutra-pharmaceutical applications, it is essential to assess its hemolytic activity. According to the results of present study, lower hemolysis was observed in case of SCFE-EO (23.55 %) as compared to HD-EO (36.94 %) supporting that the framer method based oil is relatively safer. No report is available in the literature with which we can compare the results of our present analysis.

**Table 5:** Other biological activities of *Mentha piperita* EOs extracted by different methods.

Bioactivity	Microbe	Peppermint EO		Rifamacin	Triton-x-100
		SCFE	Hydro- distillation		
**Biofilm Inhibition %**	***S. aureus***	72.32 ± 2.81^a^	61.23 ± 1.89^b^	87.43 ± 3.89^a^	–
	***E. coli***	74.57 ± 2.39^a^	33.65 ± 0.48^b^	88.92 ± 3.45^a^	–
**Hemolytic Assay %**		23.55 ± 0.92^b^	36.94 ± 1.32^a^	–	100

Result is presented as mean of three replicate measurements ± standard deviation. The significant difference (*p* < 0.05) between two extraction techniques is represented by superscripts within the same column.

The findings of our present research work conclude that the EO obtained by both the techniques were found to be a good source of potent bioactive components and hence possessed significant biological activities including antioxidant, antimicrobial and biofilm inhibition with low magnitude of hemolysis and thus can be safely used in the food industry and nutra-pharmaceutical preparations. A comparative evaluation of the present data support that SCFE is a superior technique than HD for extraction of biologically active better quality essential oil for food and pharmaceutical applications.

## 6 Conclusions

The peppermint (*Mentha piperita*) essential oil prepared by HD and SCFE methods was compared in terms of yield, chemical composition, physicochemical analysis, antioxidant activity, antibacterial activity, biofilm inhibition, and hemolytic activity. The yield of EO obtained by HD was significantly higher (*p* < 0.05) than that of SCFE with negligible variation in physical parameters like color, solubility, density (25 °C) and refractive index (25 °C) of the recovered oils. The results of the compositional analysis showed that a total of 14 components were produced by SCFE and 12 by HD. The most important component in the extracted oil obtained by both methods was menthol, which was followed by eucalyptol and menthone. In comparison to HD-EO, the SCFE-EO demonstrated greater (*p* < 0.05) antioxidant activity in terms of DPPH radical scavenging activity and reducing power. When tested against several bacteria, SCFE-EO demonstrated the strongest antimicrobial efficacy. The result of biofilm inhibition and hemolytic activity revealed that SCFE-EO is superior to HD-EO with high biofilm inhibition and lower hemolysis. In conclusion, due to its potent antioxidant and antibacterial properties, peppermint EO derived via the SCFE process can be a useful ingredient in the functional food and nutraceutical industries.

## CRediT authorship contribution statement

**Ali Abbas:** Writing – original draft, Formal analysis. **Farooq Anwar:** Writing – review & editing. **Naveed Ahmad:** Writing – review & editing. **Afifa tur Rehman:** Formal analysis, Data curation. **Osama A. Mohammed:** Resources, Project administration. **Mustafa Ahmed Abdel-Reheim:** Resources, Data curation. **Munawar Iqbal:** Writing – review & editing. **Shahid Iqbal:** Writing – review & editing. **Arif Nazir:** Methodology.

## Declaration of competing interest

The authors declare that they have no known competing financial interests or personal relationships that could have appeared to influence the work reported in this paper.
